# Efficient Egress of Escaping Ants Stressed with Temperature

**DOI:** 10.1371/journal.pone.0081082

**Published:** 2013-11-29

**Authors:** Santiago Boari, Roxana Josens, Daniel R. Parisi

**Affiliations:** 1 Departamento de Física, Facultad de Ciencias Exactas y Naturales, Universidad de Buenos Aires, Buenos Aires, Argentina; 2 Grupo de Estudio de Insectos Sociales, Facultad de Ciencias Exactas y Naturales, Universidad de Buenos Aires, Buenos Aires, Argentina; 3 Consejo Nacional de Investigaciones Científicas y Técnicas, Buenos Aires, Argentina; 4 Instituto Tecnológico de Buenos Aires, Buenos Aires, Argentina; University of Sheffield, United Kingdom

## Abstract

In the present work we investigate the egress times of a group of Argentine ants (*Linepithema humile*) stressed with different heating speeds. We found that the higher the temperature ramp is, the faster ants evacuate showing, in this sense, a group-efficient evacuation strategy. It is important to note that even when the life of ants was in danger, jamming and clogging was not observed near the exit, in accordance with other experiments reported in the literature using citronella as aversive stimuli. Because of this clear difference between ants and humans, we recommend the use of some other animal models for studying competitive egress dynamics as a more accurate approach to understanding competitive egress in human systems.

## Introduction

The study of pedestrian egress through narrow doors in life-and-death situations has huge importance in order to design safe and efficient evacuation systems.

The process we want to address is the room evacuation problem, which corresponds to an indoor area with a single narrow door and with a random initial occupation of agents that generates a uniform low-medium density over the whole area. At a given moment a hazard appears and all agents want to egress simultaneously, which can generate a high density area near the exit door.

This problem has been addressed by means of computer simulation models such as the Social Force Model (SFM) [1] and cellular automata models [2]. However, none of these models have been yet experimentally validated for systems with narrow exits and a large number of people with an urgent need to egress.

Simulations of the room evacuation problem presented in Ref. [1] show the "Faster is Slower" (FIS) effect. A key ingredient is the assumed behavior of simulated agents: they take direct paths toward the door. The FIS effect indicates that for desired velocities of simulated agents greater than a certain threshold, the mean egress time increases (the flow rate is slower) when the desired velocities increase. In other words, the faster agents want to egress, the slower they achieve it. This effect was studied by Parisi and Dorso [3] and it was shown that it appears due to tangential friction of particles in contact jamming at the door area. Experimental validation of the FIS effect for evacuating humans is a pending matter. However, it sounds reasonable because it has been observed experimentally for granular matter, considering a bi-dimensional hopper over an inclined plane [4]. In this experiment, variations in the plane angle imply variations in the acceleration of particles mimicking the different desired velocities in the SFM simulations.

Because of the difficulties arising from human experiments, it is natural to propose working with animals. Ants have been chosen by some authors, since they are relatively easy to manipulate and, in principle, share certain characteristics with humans, i.e., they are biological agents, they have different communication channels, they have a particular signal for alarm situations, etc. However, after working with two ant species and with different aversive stimuli, we found that ants have a different behavior than human beings under threatening conditions.

### 1 Humans

Valuable empirical data can be obtained during real catastrophes.

One example is the video recorded by security cameras in Mina/Makkah during the Hajj on 12 January 2006 [5]. In this system, crowd turbulence was observed and it could be reproduced by a version of the SFM [6]. However, these data correspond to a system with very large characteristic length (greater than 20 m) and thus, it does not give enough information in the range of narrow exits.

On the other hand, another documented disaster, which does show limited-space effects, was the one at "The Station Night Club" fire (Rhode Island, U.S.A.­, 20 February 2003) [7]. The evacuation process was recorded by an amateur camera and can be seen in Ref. [8]. At the beginning, only a fraction of people realized that a dangerous situation was taking place. Between 30 seconds and 1 minute after the fire started, the way out was difficult: there was friction between people because the capacity of the escape routes began to be exceeded. In this period it can clearly be observed that people who were aware of the existence of danger immediately began to walk directly toward the exit. At 1 minute and 35 seconds after the fire started, people trying to leave caused a total blockage of the exit. This blockage was due to the fact that everyone realized that a real danger was present and tried to escape simultaneously using the main entrance/exit in a direct path exceeding the capacity of the narrow exit and thus blocking it permanently.

Based on this observation, the assumption made for the behavior of pedestrians in the room evacuation process by Helbing et al. [1] seems correct.

In terms of the definition given by Soria et al. [9] this is a "selfish evacuation behavior." In the work of Heliövaara et al. [10] this behavior is named as "impatient" behavior, which can be triggered when the estimated evacuation time [11] of a given pedestrian is greater than or similar to the subjective estimation of the available safe egress time (ASET) [12].

The pedestrians' dilemma of behaving patiently or impatiently can be modeled under the scope of game theory [10] and it has been shown that under threatening conditions, jamming and clogging may be caused by people acting rationally from an individual point of view, even when this rational behavior results in a loss of evacuation performance for the whole group.

As a natural attempt to study the emergency-egress problem under controlled conditions, experiments with animals have arisen as a very first approach to obtain experimental data of biological agents that resemble human systems in some aspects.

### 2 Non-social Animals

Other animals share the "selfish evacuation behavior" with humans when aversive or positive stimuli are present.

In the experiment performed by Saloma et al. [13] 60 mice were put in a water pool and they could only escape through a narrow exit. The behavior observed was that the mice tried to reach the exit as soon as possible.

In another experiment with sheep performed by Zuriguel et al. (unpublished data), a direct path to the exit could also be observed. Sheep showed pushing and urgency to enter a room where they would be fed.

Considering the evidence presented above regarding humans and other mammals, we can state that their behavior under life-and-death situations consists in trying to avoid the danger as soon as possible going to a safe place in the most direct path.

One could assume that this “selfish evacuation behavior” is a universal constant among animal species and that it is related to the survival of the individual. However, for eusocial insects this statement is not necessarily true. Ant colonies, considered as a supraorganism, behave cooperatively prioritizing the survival of the colony over the individuals [14].

### 3 Ants

First, we want to remark that for normal conditions, the fundamental diagram of ants mismatches the one for pedestrians and vehicles. This diagram is a plot of the density versus the speed for a group of self-propelled particles. In the case of pedestrians (and vehicles) it shows a monotonically decreasing function indicating that when a pedestrian can move freely (very low density) she/he can develop her/his maximum desired velocity. However, as density increases, less free space is available and the pedestrian has to diminish her/his speed in order to avoid colliding with the precedent pedestrian. At the extreme case of very high density the velocity tends to zero. Many experimental fundamental diagrams for pedestrians can be found in the literature, see for example Refs. [5], [15], [16], [17].

On the contrary, ants have shown a constant fundamental diagram meaning that the speed of ants does not depend on the density [18]. Furthermore, ants do not produce jamming [18], [19]. One standard way of validating computer models of pedestrian movements in normal conditions is showing that the model can reproduce the fundamental diagram. The fact that this important property of pedestrian flow is not observed in ants indicates that ants do not represent a good animal model for studying pedestrian dynamics in normal conditions.

Considering emergency conditions, Altshuler et al. [20] performed an experiment with ants to study some aspects of pedestrian dynamics. In this work, the authors reported the herding behavior of ants when exposed to citronella, in contrast with individual behavior in the absence of that negative stimulus. This "follow-the-crowd" behavior was proposed as a possible behavior of simulated humans by Helbing et al. [1]. Therefore, they claim that from this point of view, the collective behavior of both organisms could be similar when escaping under emergency conditions. Maybe this is the case for this experiment; however, we would like to add that, from another point of view, both organisms can behave differently.

When stressed with citronella or high temperature (as we will show in the present work) ants do not follow a direct path to the exit and thus, they do not display any impatient or selfish evacuation behavior. This fact can be observed in several documented experiments: (a) on the web page of Ref. [20] a complete evacuation process can be seen. In that movie, jamming or clogging is not observed near the exit. (b) In Fig. 3 of Ref. [21], it can be seen that the exits do not show a higher density than the rest of the surrounding areas. (c) Finally, the fact that ants do not either jam or produce clogging in front of the exit door was reported also in a previous paper [9] for *Camponotus mus* ants. In that paper we measured a "faster is slower" (FIS) curve for escaping ants stressed with different citronella concentrations, which would represent different intensities of the aversive stimulus. However, even when the curve obtained for the evacuation time versus citronella concentration was similar to the one predicted by the FIS effect, the cause of this effect is totally different to that producing it in pedestrian simulations. As said above, in this last system the FIS is generated by strong contact and high tangential friction [3]. On the other hand, ants do not present any friction because jamming was not observed, as stated in Ref. [9]. It seems that in that experiment, the cause for the increase in the evacuation times is because ants present a movement and/or coordination performance loss, probably due to the very high citronella concentration. This last issue is under current analysis.

In the present work we will further demonstrate that ants evacuate efficiently without producing jamming or clogging upstream the exit, even with another species and under different threatening conditions than in previous work [9]. Here, we show the data corresponding to a series of experiments performed with Argentine ants (*Linepithema humile*) stressed with aversive stimuli of variable intensities given by different heating speeds of the floor, each one generating a different degree of urgency in the ants in the same way that the desired velocity is increased in the simulations showing the FIS effect [1], [3]. This means that in our experiment "faster" indicates a faster increase of the temperature. For each stimulus intensity the partial evacuation time is studied.

## Materials and Methods

### 1 Insects

We used two different colonies of the Argentine ant (*Linepithema humile*). The colonies were captured in the campus "Ciudad Universitaria" of the University of Buenos Aires in Buenos Aires City, Argentina, and transported to the laboratory. No specific permissions were required for collecting this species, which is dominant and even subject to pest control by baits in the buildings of the area.

Each colony composed of around 1000 workers and at least one queen was placed in a plastic box (20×30 cm and 20 cm high) with plaster bottom and fluon-painted walls to prevent animals from escaping. Ants nested in glass test tubes covered with metal foil placed there for that purpose. Colonies were maintained in the laboratory for a month under natural light/dark cycles and nearly constant temperature (25 ± 3 °C). The ants could move freely within the box where they had access to freshwater, honey water and commercial canned tuna in oil or commercial canned meat. For each nest, the time elapsed between two consecutive experiments was at least a week.

*Linepithema humile* is a brown or dark brown ant between 2 and 3 mm long and approximately 1 mm wide without considering its legs. Their workers are all of a similar size. Only workers were used for the experiments.

### 2 Experimental Device

**Figure 1:**
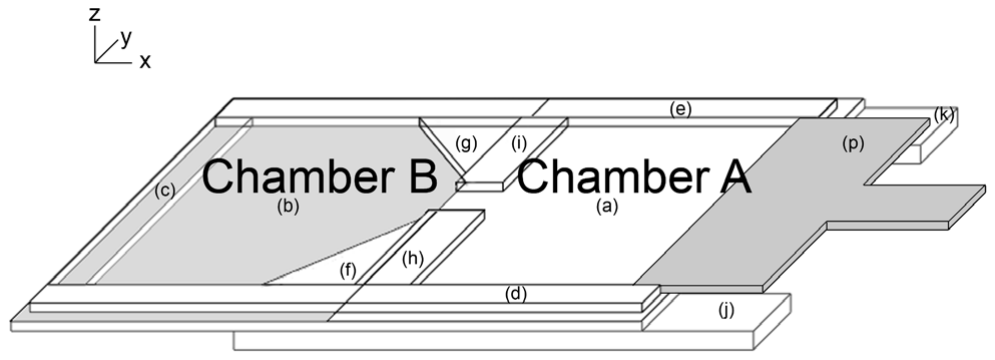
Arena scheme. The shaded area (chamber B) is the room with aluminum floor, areas (c) to (h) are a single piece of synthetic rubber and the remaining pieces are made of glass. The piston (p) is used for pushing ants to the punishable chamber. Measurements (x, y, z): (a) – (b): 14.5 cm×14 cm×2 mm, (c): 1 cm×9 cm×1.5 mm (d) – (e): 26 cm×1 cm×1.5 mm (f) – (g): 3 cm×4 cm×1.5 mm, (h) – (i): 1.5 cm×4.4 cm×1.5 mm, (j) – (k): 24 cm×2 cm×4 mm. Exit width: 2 mm.

Figure 1 shows the arena we built for our experiments, which consisted of two chambers: the complementary chamber (A) and the punishable chamber (B). Both chambers were connected by a narrow (2 mm) corridor. This width corresponded to approximately 2 times the average *L. humile* body width.

The device was built mainly of glass, except the floor of chamber B that was made of a 2 mm aluminum plate that was heated to generate the aversive stimulus. Walls were made with a unique piece of commercial rubber sheet 1 mm thick, which determined the height of the chambers. The entire setup was covered by a glass 'ceiling' to prevent ants from climbing to the walls, ceiling and on one another. This was particularly important in order to keep a 2D geometry for the experiment. In chamber A, the wall opposite the exit was a piston that allowed us to change the size of the chamber and was used primarily as a means to contain ants in chamber B during the first phase of the experimental protocol.

**Figure 2:**
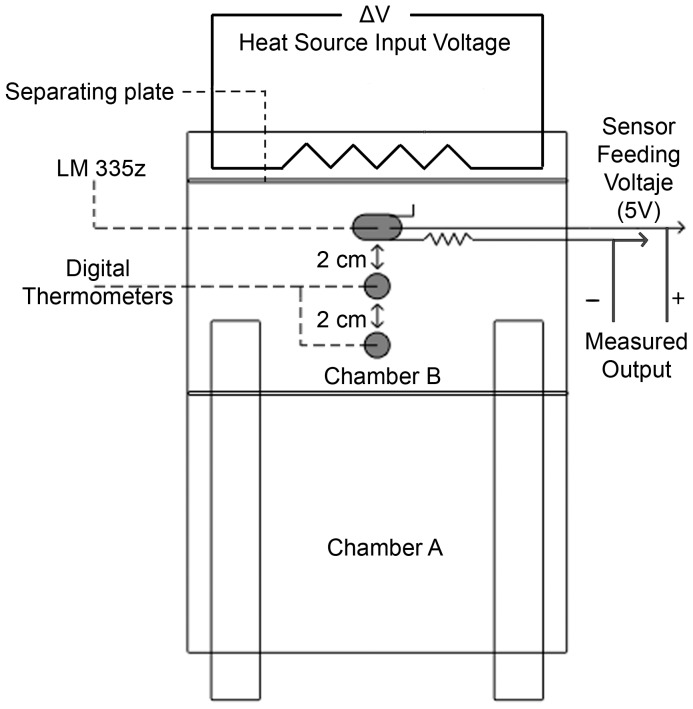
Bottom view of the arena. Positions of the heat source and the three thermometers used under the aluminum plate.

Under the aluminum floor, both the heating and temperature reading systems were placed, as shown schematically in Fig. 2.

The heat source was a dichroic lamp and its output power can be set by the input feeding voltage. This lamp is situated below chamber B, near the wall opposite the exit. Three thermometers were used to record the temperature at different sites. The thermometers were isolated from the heat source by means of an aluminum division placed in between (Fig. 2). They were attached to the aluminum floor and separated 2 cm from each other and 3 cm from the lamp. A thin layer of thermal grease was applied to the surface prior to the permanent attachment of the thermometers in order to increase the thermal conductivity of the interface between the thermometers and the aluminum plate.

Using two identical digital thermometers, it was verified before the ant trials that for any given point in the plate there were no significant differences in the temperature reading when measuring either in the top face or underside.

Ant evacuation at different heat intensities, as aversive stimulus, was studied. Intensity was given by the feeding voltage of the lamp. Each fixed voltage produced time-dependent heating of the aluminum plate and a small temperature gradient that could lead ants toward the exit. The maximum gradient measured between the two adjacent thermometers was 3°C.

**Figure 3:**
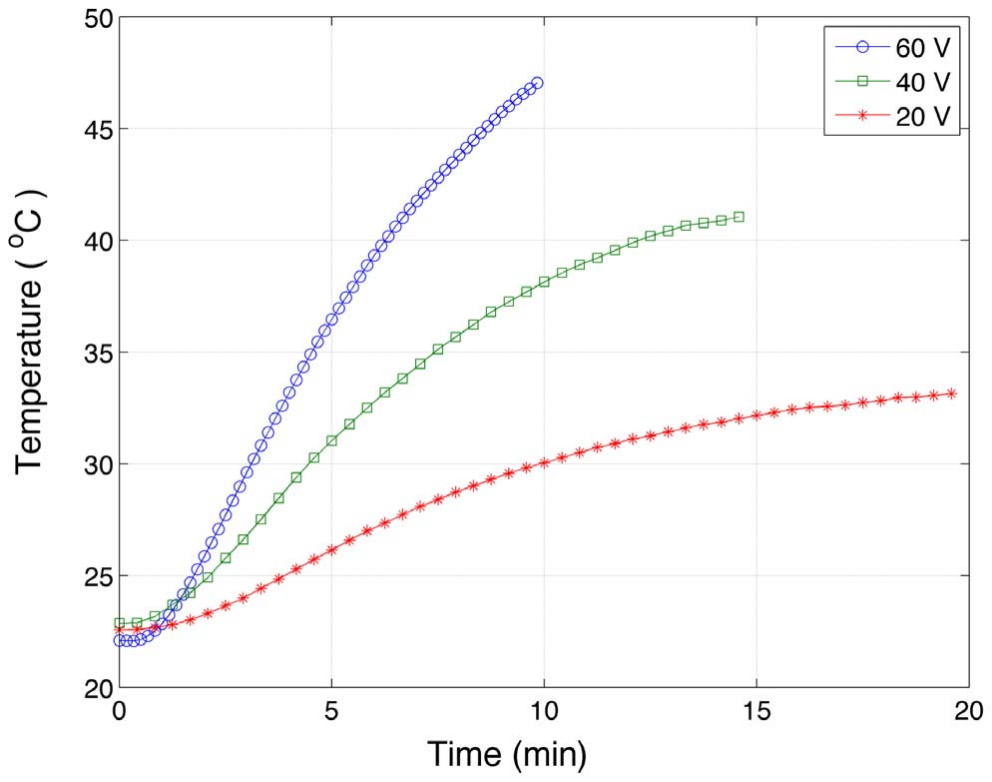
Time evolution of chamber B floor temperature. This is the punishable chamber and the temperature was measured by the integrated circuit LM335z (see Fig.2).

Typical temperature ramps measured with thermometer LM335z (see Fig. 2) are shown for different input voltages of the heating source in Fig. 3.

### 3 Experimental Protocol

The protocol for each experimental trial was the following:

- All experiments started at room temperature. At the beginning of each trial, the piston was placed 11 cm away from the door to chamber B so chamber A could have its full size.

- A group of approximately 200 ants were taken from one of the nests and placed within a flask (7.5 cm diameter) with fluon-painted walls to transport the whole group of ants from the nest to the arena in one step.

- The ants were placed in chamber A by emptying the flask and immediately covering the arena with the glass lid.

- The piston of chamber A was gently pushed until it was placed about 2 cm from the passage that communicates both chambers, generating a local high density of ants. This configuration was kept for about 1 hour, allowing ants to familiarize with the arena. In this time approximately 80% of the total number of ants passed to chamber B, as it was more spacious. The piston was then gently pushed even more, forcing the remaining ants to pass to chamber B and preventing them from returning to chamber A. This entry process ensured that every single ant had passed through the door at least once before the beginning of the recording.

- After the previous steps, 160 ± 40 ants reached the punishable chamber. At this moment, the lamp was turned on at a fixed feeding voltage. Immediately, the piston was moved back to its original position leaving the exit open and hence, chamber A was made accessible to the individuals in chamber B. At this point the evacuation process started.

- The evacuation of ants from the punishable chamber (B) was recorded with a video camera (Sony HDR-SR11, 1920×1080 pixels, at 30 fps). From the videos, evacuation times were obtained for different percentages of the initial ant group (50% and 70%).

The described protocol and experimental setup was designed with the goal of studying the room evacuation problem (defined in Section 1). Key phenomena of this process are jamming and clogging near the door. In order to force ants to achieve this condition, the following characteristics of the setup and protocol are very important:

-By initially placing the ants in chamber A and then gently pushing them to chamber B, we ensure that all of them have gone through the exit at least once, which allows them to get acquainted with the arena.

- The condition of beginning the punishment 1 hour later and keeping the arena at room temperature during this period generates an initial state of non-stress. This assures that the only perturbation is the controlled aversive stimuli.

- The position of the heat source and the fact that it is turned on at the beginning of the punishment phase of the trial generate a temperature gradient that indicates ants the direction to the door, so ants can be guided toward the door when the temperature begins to rise.

### 4 Experimental Series

Experimental data acquisition started in November 2011 and ended in April 2012.

Two control trials were made:

- 0 V control: trial made with the same protocol but with the heating source turned off (0 V).

- 60 V control: trial made with the same protocol but at input voltage 60 V.

The evacuation series consist of trials at different feeding voltages to generate different intensities of the aversive stimulus. Five different input voltages were evaluated: 15 V (4 trials), 20 V (8 trials), 30 V (7 trials), 40 V (7 trials) and 50 V (5 trials).

In total, thirty-three trials were performed.

## Experimental Results

### 1 Control Series

In the control series at 0 V, the heating source was not turned on, so the plate was at room temperature. In this condition, ants displayed low mobility and the time required for the migration of 50% of the ants to chamber A was about 150 minutes. This represents a very long characteristic time when compared to the ones obtained in the experiments at nonzero voltage (about 10 minutes for 15 V). Therefore, this control confirmed the aversive effect of temperature rise in the punishable chamber.

The control series at 60 V showed that the maximum temperature supported by ants (about 45° C) was reached very rapidly compared to the group reaction time, causing the death of about 60% of the ants participating in this run. Taking this into account, the maximum input voltage used in the rest of the trials was 50 V, which caused a mortality of less than 10%.

### 2 Evacuation Series

First, we present the results and analysis corresponding to the trials under the most extreme conditions (50V) that maximize the probabilities of obtaining the effects and behaviors we want to study.

**Figure 4:**
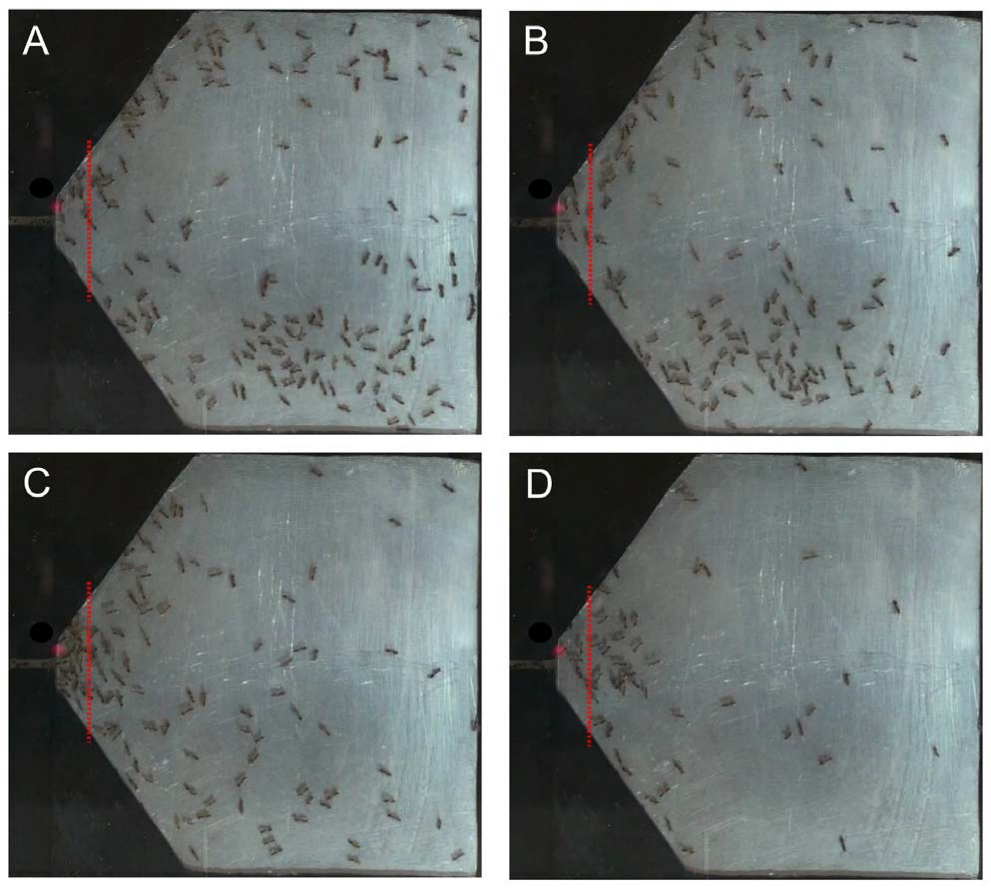
Snapshots of a fast evacuation process. Input voltage was 50 V. Frames at time (A**)** 2 min 11 s, (B) 3 min 10 s, **(C)** 4 min 25 s, (D) 5 min 30 s. The red-dashed line at 0.75 cm from the door limits the area where the density shown in Fig.5 B is measured.

**Figure 5:**
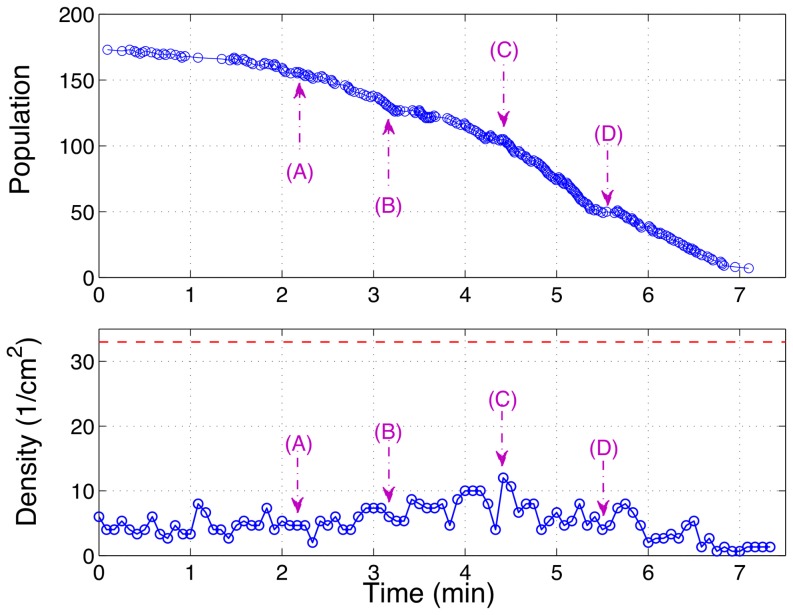
Evolution of population and density for the same trial shown in Fig. 4. (A) Population inside the punishable chamber as a function of time. (B) Evolution of the density near the door in the area of 1.5 cm^2^ limited by the walls and the red-dashed line in Fig. 4. The red line at 33 ants/cm^2^ is the approximated maximum achievable ant density considering an ant body of 0.03 cm^2^ in a close packing. In both panels the arrows indicate the frames of Fig. 4.

An evacuation process corresponding to one of the quickest evacuations can be observed in Fig. 4. A vertical dashed line is drawn at 0.75 cm from the door, which limits an area of 1.5 cm^2^ bounded also by the walls and the door line. This area is used to characterize the density of ants near the door for this trial as shown in Fig. 5. This figure also displays the discharge curve, for the same trial, which allows one to see the evolution of the ant population inside the punishable chamber as a function of time. The frames of Fig. 4 (A, B, C and D) were selected at different times of the evacuation process considering medium and maximum densities indicated as arrows in both time series of Fig.5.

**Figure 6:**
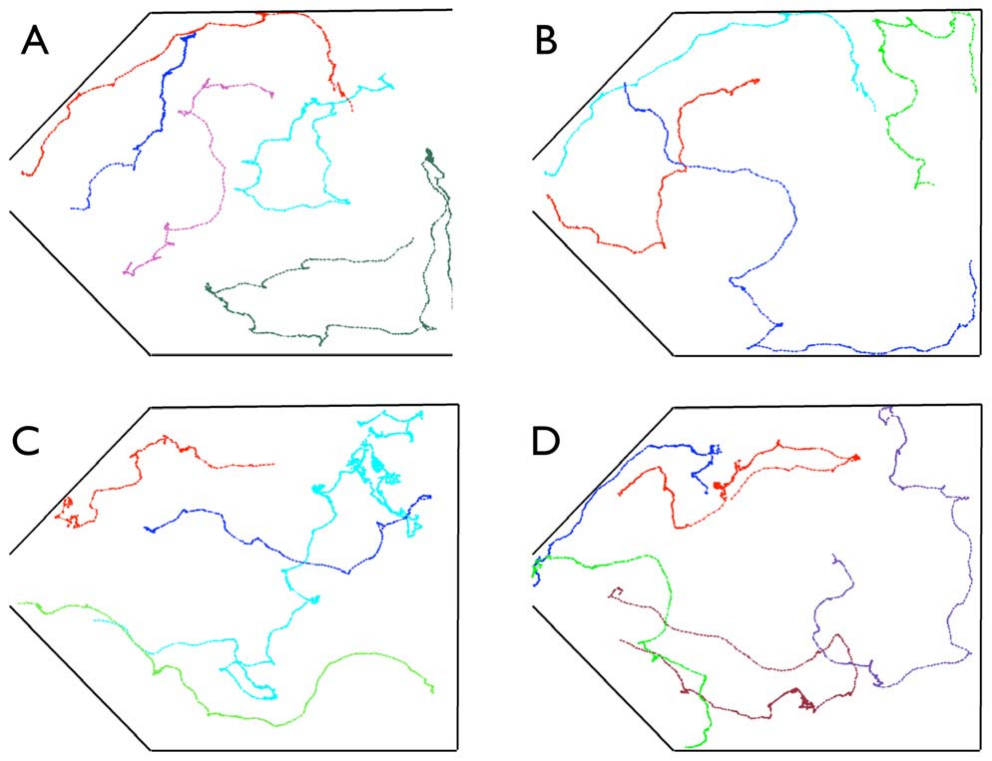
Individual Trajectories. The 4 panels show 4 arbitrary sets of randomly chosen trajectories during one of the quickest evacuation process (same trial as in Fig. 4 and 5).

Using image processing technics [22] individual trajectories can be obtained from the video and several randomly chosen ones are presented in Fig. 6.

From information expressed in Figs. 4 – 6, it can be observed that for this trial corresponding to the most extreme conditions (50V) no "selfish evacuation behavior" is displayed by ants. This statement is justified below:

- If this behavior were present, the ants would occupy all the available area at the beginning of the trial, but after a short time all of them would be jammed near the door trying to exit by pushing each other during all the rest (being the major part) of the evacuation, and consequently all the frames shown in Fig. 4 would display only a very high density of ants at the door and the rest of the area without ants. Clearly this is not the case.

- From the orientation of the bodies of ants the direction of their velocities can be inferred and it can be seen that they are distributed in several directions. Again, this observation differs from a "selfish evacuation behavior" in which all ant velocities should point towards the door. Furthermore, in Fig. 6 it can be observed that ants do not move in a direct path to the door.

- The frame in Fig. 4 (C) shows the maximum density near the door observed during the trial (12 ants/cm^2^) and it can be seen that high contact between ants is absent even in this extreme case. Also, Fig. 5 (B) shows that this maximum density is just a singular event given that the mean density of this trial is 5.1 ants/cm^2^ (∼ 15% of the theoretical maximum density of 33 ants/cm^2^ drawn as dashed line). If "selfish evacuation behavior" were present, the density should be much greater and last almost all the evacuation process consistently.

Having analyzed in detail one trial corresponding to the maximum intensity of the stimulus, we now present the results from the whole set of the trials performed. From the videos recorded, the discharge curves can be obtained and thus, the evacuation times for different fractions of the ant population can be measured.

**Figure 7:**
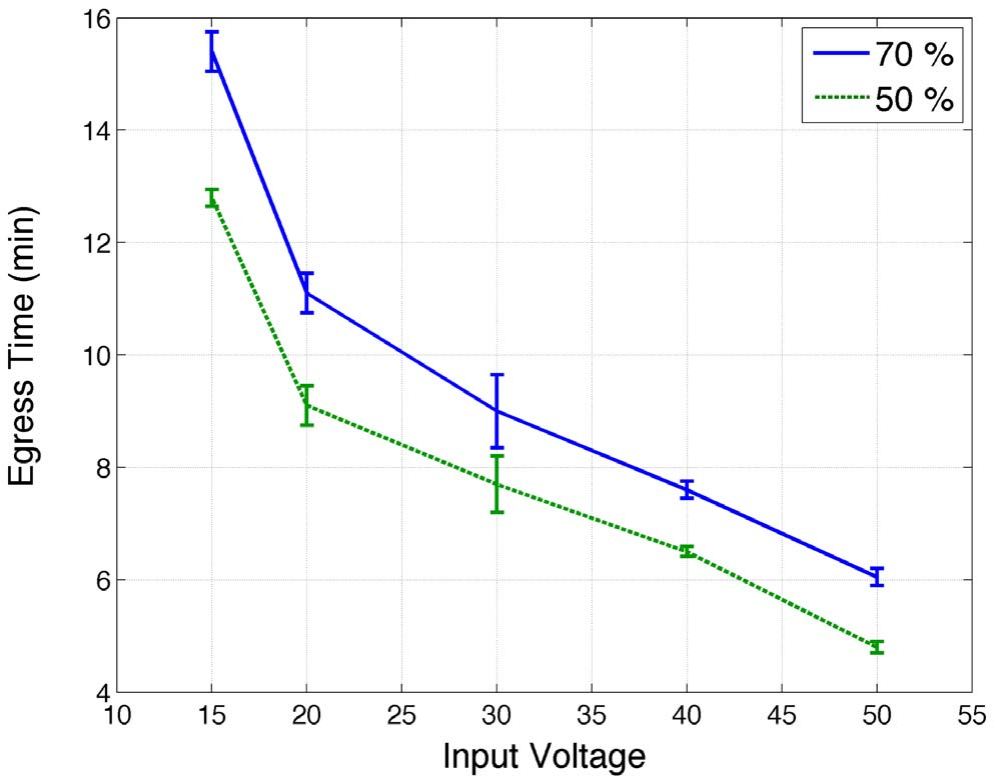
Mean egress time. Evacuated fractions (50% and 70%) of the initial population versus the input voltage of the heating source. Error bars correspond to the standard error of the mean.

For each trial, the input voltage of the heating source is fixed, generating the temperature evolution and gradient described in Section 2.2. Thus, this voltage was taken as the control variable of the experiments and so the partial evacuation times are plotted against the input voltage in Fig. 7.

In spite of the variations in the initial population of ants, the behavior of the different fractions of egressed ants turned out to be consistent, i.e., they showed very similar evacuation times, as can be seen from error bars in Fig. 7.

The evacuation time decreased monotonically with the intensity of the aversive stimulus (Fig. 7). In other words, the faster the ants need to evacuate, the faster they do it, indicating that ants perform an efficient egress process.

This is in clear contradiction to the FIS effect predicted by simulations [1] and observed in granular flows [4].

A necessary condition for this effect to occur is having strong contact and friction between particles [3]. This condition is not observed in ants, as can be seen in Figs. 4 and 5 for a trial with the maximum intensity of the aversive stimuli studied, corresponding to the faster increase of the temperature in the punishable chamber.

Summarizing, ants do not show a "selfish evacuation behavior" during the experiments performed. They do not follow direct trajectories towards the exit. By doing this, blocking clusters and clogging at the exit are avoided maintaining a fluid evacuation at all times. The absence of jamming upstream the exit is consistent with the results of a similar experiment with another ant species and another aversive stimulus [9], with the data reported by Altshuler et al. [20] and with Shiwakoti et al. [21] as described in Section 1.3

## Discussion

It has already been stated that ants do not exhibit a "selfish evacuation behavior" when stressed with citronella [9]. An objection could be that citronella is unpleasant but maybe does not endanger their lives. However, the high temperature clearly endangers their lives and ants showed the same result: they do not jam either with citronella or with high temperatures.

All ant species are eusocial insects, i.e., they have a social life in a colony with separate roles among members, with a reproductive caste and a more numerous sterile caste, the workers (which are all female). Contrary to most animals, the reproductive unit is the colony rather than the individual ant. Rules of individual decision-making are essential in social insects, for which cooperation is fundamental for the emergence of coordinated behavior. Ants and honeybees are useful biological models for studying collective decision-making processes in group-living animals. These insect societies, even when composed of thousands of individuals, behave coordinately as a whole [23], [24]. As ants used in the mentioned experiments are of the same colony, they should be expected to act more cooperatively than pedestrians in danger.

The ants studied in the present work and in others [9], [20], [21] do not show jamming or clogging upstream the exit. Considering this and what is stated in Section 1.1, ants and humans do not seem to behave in the same way either in normal conditions or in the case of egress under highly competitive situations (when the available time to escape is very short). Therefore, the methodology proposed by Shiwakoti et al. [21] and Shiwakoti and Sarvi [25] could lead to serious misunderstandings of human emergency egress. In those papers the authors claim that the way ants behave under emergency conditions is equivalent to how humans behave under such situations. Following this strong premise, the authors performed ant experiments, adjusted the experimental data to a variation of the SFM, fitted the relevant ant parameters, scaled them up for the human case and proposed using this model to make predictions in order to design human evacuation systems. We would like to emphasize that as the premise was not demonstrated to be true, the predictions for human systems are therefore unreliable and thus, they should not be used for designing real pedestrian facilities.

Following the results presented, we consider that no human egress model can be "validated" under emergency conditions by using the results of ant experiments. This important finding indicates that other animals should be used accordingly, in order to have a system more similar to people in a state of competitive egress. Even in the case of using animals showing "selfish evacuation behavior", the similarities and equivalence to human systems should be supported and demonstrated by quantitative data before drawing conclusions about pedestrian evacuation dynamics.

Future work will process all the obtained images to gain a better understanding of trajectories, velocities and what ants do individually when escaping in order to evacuate efficiently. This knowledge will be worth in itself, but it could also shed light on how to apply it in order to improve the egress of humans under threatening conditions.

## Conclusions

In the present work we study the egress of ants stressed with different heating speeds.

By studying one of the trials corresponding to the maximum intensity of the aversive stimulus, we show that the "selfish evacuation behavior" is not displayed by ants, which avoids jamming and clogging near the exit.

We also found that the mean evacuation time decreases monotonically with increasing temperature ramps. This indicates that ants evacuate efficiently independently of the degree of hurry, which can be named as the "Faster is Faster" effect in contradiction with the "Faster is Slower" effect expected for humans and most of the animal species.

Because of this great difference, we recommend not using ant experimental data for the design of humans egress systems. We propose, instead, using any other animal species that share more behavioral resemblances with humans as a very first approach to the study of human systems based on animal models.
